# Pannexin 1-Mediated ATP Signaling in the Trigeminal Spinal Subnucleus Caudalis Is Involved in Tongue Cancer Pain

**DOI:** 10.3390/ijms222111404

**Published:** 2021-10-22

**Authors:** Ryo Koyama, Koichi Iwata, Yoshinori Hayashi, Suzuro Hitomi, Ikuko Shibuta, Akihiko Furukawa, Sayaka Asano, Tadayoshi Kaneko, Yoshiyuki Yonehara, Masamichi Shinoda

**Affiliations:** 1 Department of Oral and Maxillofacial Surgery II, Nihon University School of Dentistry, 1-8-13 Kandasurugadai, Chiyoda-ku, Tokyo 101-8310, Japan; dery18014@g.nihon-u.ac.jp (R.K.); furukawa.akihiko@nihon-u.ac.jp (A.F.); kaneko.tadayoshi@nihon-u.ac.jp (T.K.); yonehara.yoshiyuki@nihon-u.ac.jp (Y.Y.); 2 Department of Physiology, Nihon University School of Dentistry, 1-8-13 Kandasurugadai, Chiyoda-ku, Tokyo 101-8310, Japan; iwata.kouichi@nihon-u.ac.jp (K.I.); hitomi.suzuro@nihon-u.ac.jp (S.H.); shibuta.ikuko@nihon-u.ac.jp (I.S.); asano.sayaka@nihon-u.ac.jp (S.A.); shinoda.masamichi@nihon-u.ac.jp (M.S.)

**Keywords:** microglia, pannexin 1, adenosine triphosphate, squamous cell carcinoma, tongue cancer pain

## Abstract

Pain is one of the most severe concerns in tongue cancer patients. However, the underlying mechanisms of tongue cancer pain are not fully understood. We investigated the molecular mechanisms of tongue cancer-induced mechanical allodynia in the tongue by squamous cell carcinoma (SCC) inoculation in rats. The head-withdrawal threshold of mechanical stimulation (MHWT) to the tongue was reduced following SCC inoculation, which was inhibited by intracisternal administration of 10Panx, an inhibitory peptide for pannexin 1 (PANX1) channels. Immunohistochemical analyses revealed that the expression of PANX1 was upregulated in the trigeminal spinal subnucleus caudalis (Vc) following SCC inoculation. The majority of PANX1 immunofluorescence was merged with ionized calcium-binding adapter molecule 1 (Iba1) fluorescence and a part of it was merged with glial fibrillary acidic protein (GFAP) fluorescence. Spike frequencies of Vc nociceptive neurons to noxious mechanical stimulation were significantly enhanced in SCC-inoculated rats, which was suppressed by intracisternal 10Panx administration. Phosphorylated extracellular signal-regulated kinase (pERK)-immunoreactive (IR) neurons increased significantly in the Vc after SCC inoculation, which was inhibited by intracisternal 10Panx administration. SCC inoculation-induced MHWT reduction and increased pERK-IR Vc neuron numbers were inhibited by P2X7 purinoceptor (P2X7R) antagonism. Conversely, these effects were observed in the presence of P2X7R agonist in SCC-inoculated rats with PANX1 inhibition. SCC inoculation-induced MHWT reduction was significantly recovered by intracisternal interleukin-1 receptor antagonist administration. These observations suggest that SCC inoculation causes PANX1 upregulation in Vc microglia and adenosine triphosphate released through PANX1 sensitizes nociceptive neurons in the Vc, resulting in tongue cancer pain.

## 1. Introduction

Squamous cell carcinoma (SCC) is one of the most common cancers in the intraoral structures, oral mucosa and tongue and it destroys the intraoral mucosa, gingiva and tongue tissues with its invasion [1]. SCC growth in the oral cavity causes tissue destruction and damage to the trigeminal nerve and local inflammation in the oral structures during tumor growth causes a deficit in oral functions, such as chewing and swallowing [2]. One of the most severe concerns in cancer patients is pain and this is a critical factor that reduces quality of life (QOL). Consequently, pain control is an important issue for improving QOL; however, the pathogenic mechanisms of tongue cancer pain have not been fully elucidated. Studies have shown that trigeminal nerve injury caused by the tumor leads to persistent neuropathic pain in a broad area of the orofacial regions [3]. An animal model study reported that SCC cells can release several molecules, such as cytokines and endothelin [4], which contribute to the enhancement of trigeminal ganglion (TG) neuronal excitability [5]. SCC in the tongue causes several changes not only in TG neurons but also in the trigeminal spinal subnucleus caudalis (Vc), where it activates microglia [6]. Although the changes in the central nervous system (CNS) caused by cancer are progressively evident, CNS changes caused by SCC are not fully understood.

Pannexin 1 (PANX1) is a large pore membrane channel that is responsible for nonselective ion permeability and the extracellular release of signal molecules, such as adenosine triphosphate (ATP), glutamate, interleukin (IL)-1β, tumor necrosis factor-α and arachidonic acid [7]. PANX1 expression is observed in microglia in the spinal cord, which contributes to chronic joint pain [8] and morphine withdrawal [9]. Such activation of microglia is accompanied by morphological activation and activated microglia are seen in the Vc during tongue cancer in rats [6]. Thus, we hypothesized that PANX1 in Vc microglia is involved in the development of tongue cancer pain. The purpose of this study was to investigate the contribution of PANX1 to nociception in tongue cancer.

## 2. Results

### 2.1. PANX1 Is Involved in the Development of Mechanical Allodynia in the Tongue following SCC Inoculation

**Figure 1:**
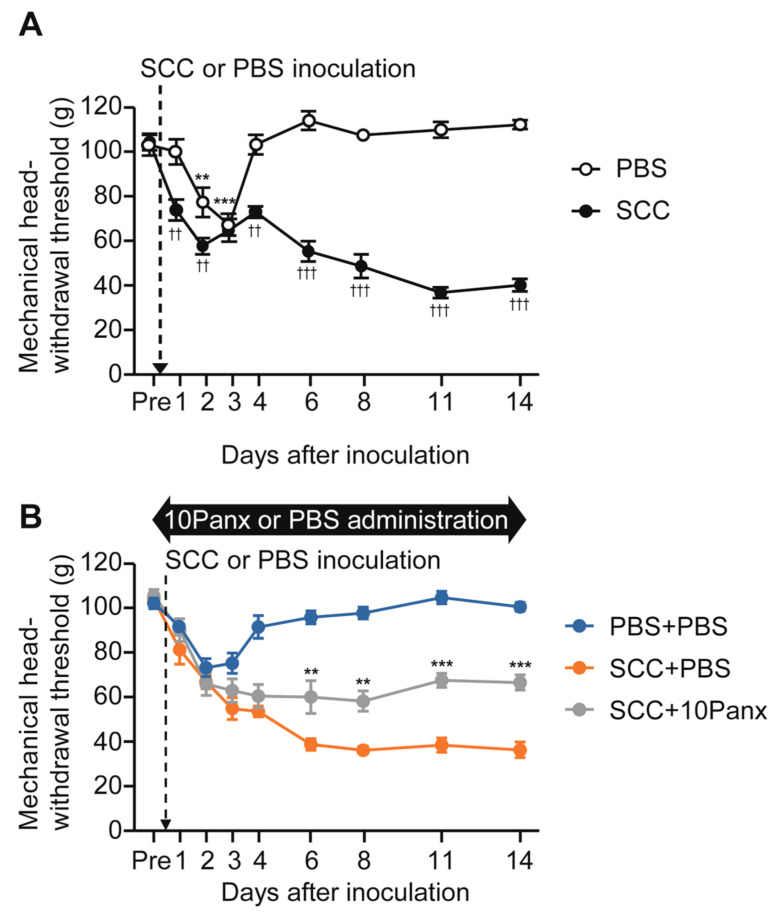
The possible involvement of PANX1 in the Vc in mechanical allodynia in the tongue following SCC inoculation. (**A**) Time course of mechanical head-withdrawal threshold with stimulation of the tongue in PBS-injected or SCC-inoculated rats. *n* = 8: PBS, *n* = 12: SCC, ** *p* < 0.01, *** *p* < 0.001, vs. Pre, one-way ANOVA post hoc Dunnet’s test; †† *p* < 0.01, ††† *p* < 0.001, vs. PBS, two-way repeated-measures ANOVA followed by Bonferroni’s test. Data represent the mean ± SEM. (**B**) Time course of mechanical head-withdrawal threshold with stimulation of the tongue in PBS-injected or SCC-inoculated rats with intracisternal administration of PBS or 10Panx. *n* = 9: PBS + PBS, *n* = 6: SCC + PBS, *n* = 10: SCC + 10 Panx, ** *p* < 0.01, *** *p* < 0.001, vs. SCC + PBS, two-way repeated-measures ANOVA followed by Bonferroni’s test. Data represent the mean ± SEM.

To analyze the role of PANX1 in the development of mechanical allodynia in the tongue following SCC inoculation, we first checked whether tongue SCC caused mechanical allodynia in the tongue. The mechanical head-withdrawal threshold (MHWT) was temporarily reduced on days 2 and 3 following phosphate-buffered saline (PBS) injection in the tongue; however, it recovered to baseline level on day 4 (Figure 1A). In contrast, MHWT in SCC-inoculated rats showed a gradual but significant reduction throughout the experimental period (Figure 1A).

To evaluate the possible involvement of PANX1 in the Vc in the development of mechanical allodynia following SCC inoculation, we administered 10Panx (an inhibitory peptide for PANX1 channels) intracisternally (i.c.) prior to SCC inoculation. Intracisternal PBS had no significant effects on MHWT in PBS-injected or SCC-inoculated rats (Figure 1A,B). On the contrary, i.c. 10Panx significantly inhibited the reduction of MHWT following SCC inoculation compared to i.c. PBS (Figure 1B).

### 2.2. Expression Patterns of PANX1 in the Vc

**Figure 2:**
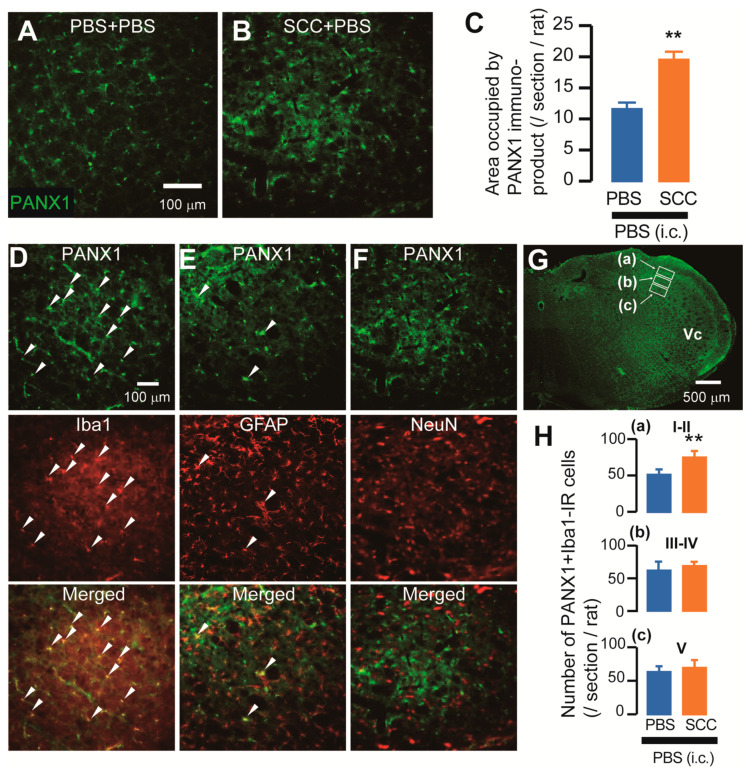
Expression of PANX1 in the Vc. (**A**,**B**) PANX-IR cells in the Vc 14 days after PBS injection with intracisternal (i.c.) administration of PBS (PBS + PBS) or SCC inoculation with PBS (i.c.) (SCC + PBS). Scale bar = 100 μm. (**C**) Columns represent the average values of area occupied by PANX1 immuno-product. *n* = 5: PBS + PBS, *n* = 5: SCC + PBS, unpaired Student’s *t*-test, ** *p* < 0.01. Data represent the mean ± SEM. (**D**–**F**) Representative images showing PANX1 (green) and Iba1, GFAP, or NeuN (red) in the Vc. Arrowheads indicate PANX1/Iba1 or PANX1/GFAP double-positive cells. Scale bar = 100 μm. (**G**) Representative PANX1 image in the Vc. Insets were set in the lamina I-II (a), III-IV (b) and V (c). Scale bar = 500 μm. (**H**) Columns represent the average number of PANX1/Iba1 double-positive cells in each lamina. a–c correspond to the inset in (**G**). *n* = 5: PBS + PBS (i.c.), *n* = 5: SCC + PBS (i.c.), unpaired Student’s *t*-test, ** *p* < 0.01. Data represent the mean ± SEM.

The expression of PANX1 in the Vc was analyzed after PBS injection or SCC inoculation in the tongue. On day 14 after PBS injection or SCC inoculation, PANX1-immunoreactive (IR) cells were observed in the Vc (Figure 2A,B). The density of PANX1-IR cells in the Vc of SCC + PBS rats was significantly higher than that in the Vc of PBS + PBS rats (Figure 2C). The localization of PANX1 in the Vc of SCC-inoculated rats was further analyzed. The majority of PANX1-IR cells were merged with ionized calcium-binding adapter molecule 1 (Iba1, a marker of microglia)-IR cells and a part of it was merged with glial fibrillary acidic protein (GFAP, a marker of astrocytes)-IR cells (Figure 2D,E). However, no PANX1-IR cells were merged with neuronal nuclei (NeuN, a marker of neurons)-IR cells (Figure 2F). The expression pattern of PANX1 was further examined. The number of PANX1-IR cells in the dorsal portion of the Vc (3rd branch region) was significantly more in SCC-inoculated rats than in PBS-inoculated rats, whereas that in 1st and 2nd branch regions was unaltered by SCC inoculation (Figure 2G). Increased number of Iba1 and PANX1-IR cells in the dorsal portion of the Vc following SCC inoculation was restricted in the lamina I-II (Figure 2H).

### 2.3. PANX1 Contributes to the Enhancement of Firing Activity in the Vc Neurons following SCC Inoculation

**Figure 3:**
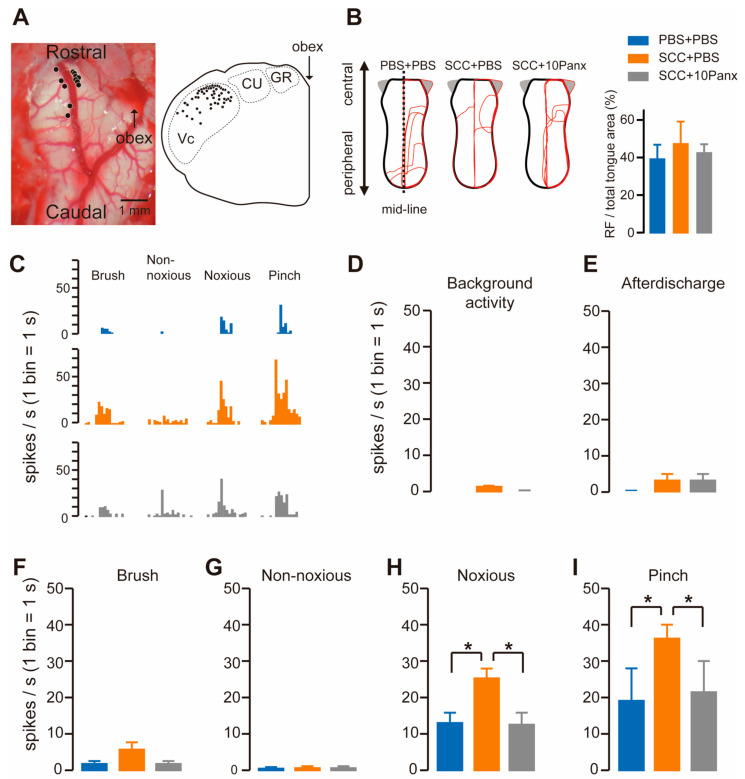
PANX1 is required for the enhancement of firing activity in Vc neurons following SCC inoculation. (**A**) Representative image showing the brainstem. An illustration showing the transverse plane of the brainstem. The black circles indicate the area that responds to the tongue stimulation. (**B**) The RFs of the tongue 14 days after PBS injection with intracisternal (i.c.) administration of PBS (PBS + PBS), SCC inoculation with PBS (i.c.) (SCC + PBS), or SCC inoculation with 10Panx (i.c.) (SCC + 10Panx). The area surrounded by the red lines indicates the area that responds to the stimulus. Columns represent the average ratio of RF in the tongue. (**C**) Representative firing activities following stimulation with brush, non-noxious, noxious and pinch stimulation in PBS + PBS, SCC + PBS and SCC + 10Panx rats. (**D**–**I**) Columns represent the average number of spikes of background activity (**D**) and afterdischarge (**E**) and brush (**F**), non-noxious pressure (**G**), noxious pressure (**H**) and pinch (**I**) induced spikes in PBS + PBS, SCC + PBS and SCC + 10Panx rats. *n* = 5: PBS + PBS, *n* = 6: SCC + PBS, *n* = 4: SCC + 10Panx, * *p* < 0.05, one-way ANOVA followed by Tukey’s test. Data represent the mean ± SEM.

To check whether Vc neurons were activated following SCC, we recorded firing activity in the dorsal portion of the Vc by inserting an electrode. The recording site was marked by applying direct currents through the electrode, which were plotted in the transverse plane of the brainstem image. The black circles show the penetration tracks where nociceptive neurons receiving noxious input from the tongue were encountered in the Vc (Figure 3A). The receptive fields (RFs) in the tongue were analyzed in PBS + PBS, SCC + PBS and SCC + 10Panx rats. However, no significant group differences were observed in terms of size (Figure 3B). All recorded neurons responded to non-noxious and noxious stimuli, indicating that they were classified as wide dynamic range (WDR) neurons (Figure 3C). Background activity and after discharge in Vc neurons were unaltered by SCC inoculation (Figure 3D,E). The mean spike frequencies of Vc nociceptive neurons after brush and non-noxious mechanical stimulation of the tongue were not different among the three groups (Figure 3F,G). In contrast, mean spike frequencies of Vc nociceptive neurons after noxious pressure and pinch stimuli were significantly larger in the SCC + PBS group than in the PBS + PBS group (Figure 3H,I). The increased firing activity in the Vc by tongue stimulation with noxious stimuli in the SCC rats was significantly suppressed by 10Panx (Figure 3H,I).

### 2.4. Inhibition of PANX1 Reduces Phosphorylation of Extracellular Signal-Regulated Kinase (ERK) in Vc Neurons following SCC Inoculation

**Figure 4:**
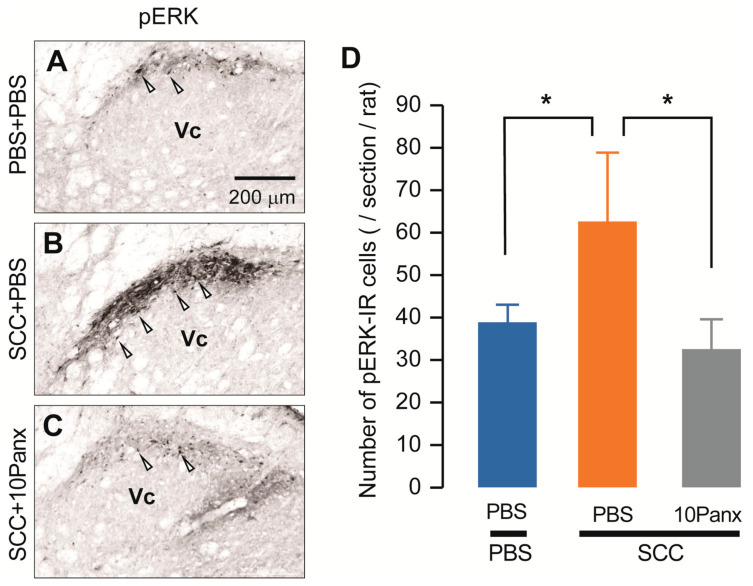
PANX1 contributes to the increment of phosphorylation of ERK in Vc neurons following SCC inoculation. (**A**–**C**) The representative images showing pERK Vc neurons in PBS + PBS, SCC + PBS and SCC + 10Panx rats on day 14 after inoculation. Scale bar = 200 μm. (**D**) Columns represent the average number of pERK-IR neurons in the Vc. Arrowheads indicate pERK-IR neurons. *n* = 5: PBS + PBS, *n* = 5: SCC + PBS, *n* = 5: SCC + 10Panx, * *p* < 0.05, one-way ANOVA followed by Tukey’s test. Data represent the mean ± SEM.

We also assessed increased neuronal activity in the Vc of SCC-inoculated rats using anti-phosphorylated extracellular signal-regulated kinase (pERK, a marker of activated neurons). On day 14 after PBS injection in the tongue, pERK-IR neurons were observed after repetitive stimulation with 60 g von Frey filament (Figure 4A). The number of pERK-IR neurons was significantly higher in SCC-inoculated rats than in PBS-injected rats (Figure 4B). In contrast, the number of pERK-IR neurons in SCC-inoculated rats administered i.c. 10Panx was significantly lower than in those administered i.c. PBS (Figure 4C,D).

### 2.5. Functional Requirement of P2X7Rs in SCC-Induced Mechanical Allodynia

**Figure 5:**
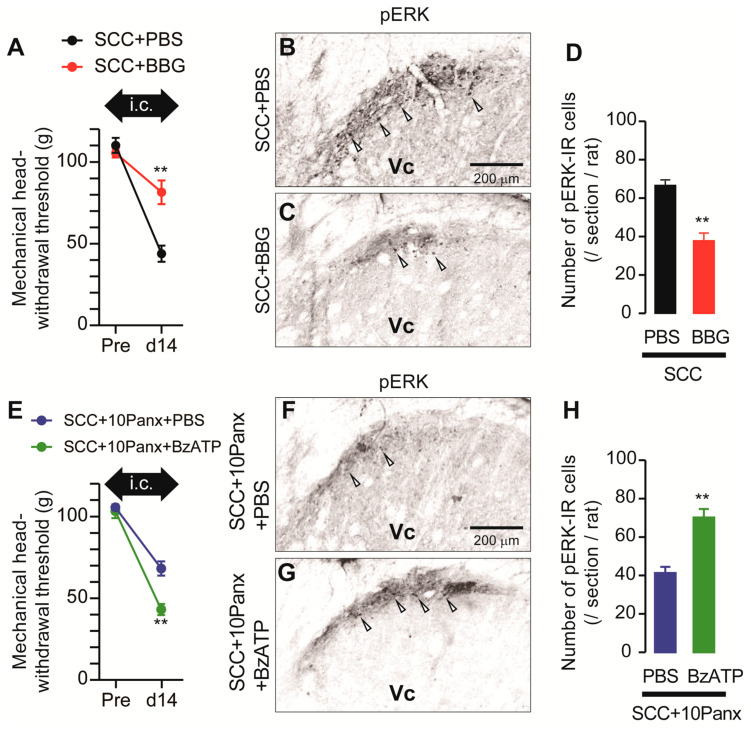
Requirement of P2X7R in the Vc in the tongue SCC-induced mechanical allodynia. (**A**) The mechanical head-withdrawal threshold in SCC-inoculated rats with intracisternal administration of PBS or BBG. The black arrow indicates the period of intracisternal administration of drugs. ** *p* < 0.01, vs. SCC + PBS, two-way ANOVA. (**B**,**C**) The representative images showing pERK Vc neurons in SCC + PBS and SCC + BBG rats on day 14 after inoculation. Arrowheads indicate pERK-IR neurons. Scale bar = 200 μm. (**D**) Columns represent the average number of pERK-IR cells in the Vc. *n* = 5: SCC + PBS, *n* = 5: SCC + BBG, ** *p* < 0.01, unpaired Student’s *t*-test. (**E**) The mechanical head-withdrawal threshold in SCC-inoculated 10Panx-administered rats with intracisternal administration of PBS or BBG. The black arrow indicates the period of intracisternal administration of drugs. ** *p* < 0.01, two-way ANOVA. (**F**,**G**) The representative images showing pERK-IR Vc neurons in SCC + 10Panx + PBS and SCC + 10Panx + BzATP rats on day 14 after inoculation. Scale bar = 200 μm. (**H**) Columns represent the average number of pERK-IR neruons in the Vc. *n* = 5: SCC + 10Panx + PBS, *n* = 5: SCC + 10Panx + BzATP, ** *p* < 0.01, vs. SCC + 10Panx + PBS, unpaired Student’s *t*-test. Data represent the mean ± SEM.

ATP is a representative molecule that is released through PANX1 [10] and P2X7Rs are representative ATP receptors that contribute to the development of mechanical allodynia [11]. We, thus, speculated that PANX1-mediated ATP secretion might be involved in the development of mechanical allodynia following SCC inoculation. To investigate the possible involvement of P2X7Rs in the development of mechanical allodynia in the tongue after SCC inoculation, Brilliant Blue G (BBG; an antagonist of P2X7Rs) was intracisternally administered prior to SCC inoculation. MHWT in SCC + BBG rats was significantly lower than that in SCC + PBS rats (Figure 5A). The number of pERK-IR Vc neurons in SCC-inoculated rats was significantly reduced by i.c. BBG (Figure 5B–D). To further analyze the requirement of P2X7Rs in mechanical allodynia in the tongue caused by tongue cancer, BzATP (a specific agonist of P2X7Rs) was intracisternally administered to SCC + 10Panx rats. The MHWT in SCC + 10Panx + BzATP rats was significantly lower than that in SCC + 10Panx + PBS rats (Figure 5E). The number of pERK-IR Vc neurons in SCC + 10Panx + BzATP rats was significantly higher than that in SCC + 10Panx + PBS rats (Figure 5F–H).

### 2.6. IL-1 Receptor Antagonism Ameliorates Mechanical Allodynia in the Tongue Caused by SCC Inoculation

**Figure 6:**
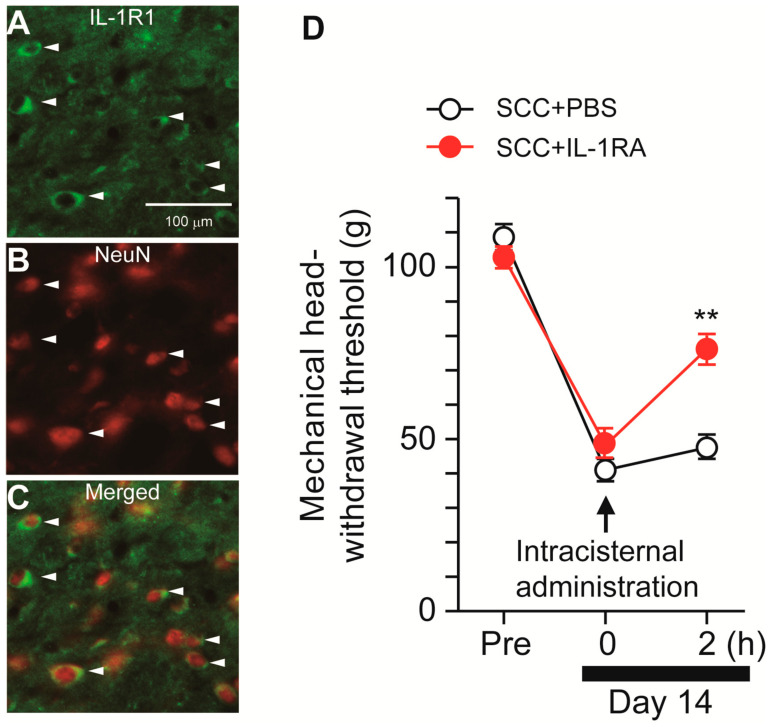
Possible involvement of IL-1β signaling in the Vc in the tongue SCC-induced mechanical allodynia. (**A**–**C**) The images showing IL-1R1 (green) and NeuN (red) in the Vc 14 days after SCC inoculation. Arrowheads indicate IL-1RI/NeuN double-positive cells. Scale bar = 100 μm. (**D**) Time course of mechanical head-withdrawal threshold in SCC-inoculated rats. PBS or IL-1RA were intracisternally administered 14 days after SCC inoculation. *n* = 5: SCC + PBS, *n* = 5: SCC + IL-1RA, ** *p* < 0.01, vs. SCC + PBS, two-way repeated-measures ANOVA followed by Bonferroni’s test. Data represent the mean ± SEM.

Following stimulation with ATP, microglia synthesize IL-1β via P2X7R-mediated activation of the nod-like receptor family pyrin domain containing 3 (NLRP3) inflammasome [12]. We, thus, speculated that microglia-derived IL-1β might contribute to SCC-induced mechanical allodynia in the tongue. The expression pattern of IL-1R1 (IL-1 receptor) in Vc was analyzed. IL-1R1-IR cells were merged with NeuN-IR cells, indicating that IL-1R1 is expressed in Vc neurons (Figure 6A–C). To investigate the contribution of IL-1β to mechanical allodynia in the tongue following SCC inoculation, IL-1RA (an IL-1 receptor antagonist) was intracisternally administered on day 14 after SCC inoculation. MHWT was significantly ameliorated by i.c. IL-1RA, but not by i.c. PBS (Figure 6D).

## 3. Discussion

The present results are summarized as follows: (1) Mechanical hypersensitivity was induced in the tongue of SCC-inoculated rats; (2) Intracisternal administration of 10Panx inhibited the development of SCC inoculation-induced mechanical allodynia in the tongue; (3) Increased neuronal activity and the number of pERK-IR Vc neurons following SCC inoculation were suppressed by i.c. 10Panx; (4) Intracistrenal administration of BBG also inhibited SCC-induced reduction of MHWT and an increase in the number of pERK-IR neurons in the Vc; (5) Intracisternal administration of BzATP caused a reduction in MHWT and an increase in the number of pERK-IR Vc neurons in 10Panx-administered SCC rats; and (6) Reduced MHWT was recovered by i.c. IL-1RA. These observations suggest that PANX1-mediated ATP secretion from microglia plays a crucial role in tongue cancer pain. 

pERK-IR neurons were observed in the restricted area of the Vc following SCC inoculation. Similarly, in vivo neuronal recordings revealed that the restricted area of the Vc responds to noxious stimulation of the tongue. These data are consistent with pERK expression appearing in a limited Vc region upon intra-tongue administration of capsaicin or tongue drying [13,14]. No significant differences in responses to non-noxious brush or pressure stimulation of the tongue between PBS-injected and SCC-inoculated rats were observed. In contrast, noxious mechanical stimulation of the tongue caused an increase in the number of pERK-IR cells in the Vc following SCC inoculation, suggesting that SCC inoculation predominantly enhances noxious but not non-noxious responses in Vc WDR neurons.

Activation of microglia in the Vc has been reported in several animal models, including trigeminal nerve injury, orofacial inflammation and SCC inoculation in the orofacial regions [6,15,16]. After cellular activation, microglia change their morphology into an activated phenotype. Along with these changes, microglia synthesize a variety of molecules [17]. ATP is a representative substance secreted by microglia and ATP release is considered to be a result of PANX1 activation [10,18]. Mechanical allodynia caused by SCC inoculation was significantly inhibited by intracisternal administration of a P2X7R antagonist. Considering the above facts, ATP secreted from microglia might directly bind to P2X7R in Vc neurons, resulting in the facilitation of ERK phosphorylation. Indeed, a robust ion influx enhances ERK phosphorylation in Vc neurons [14]. On the contrary, ATP from microglia might bind to Vc microglia according to the finding that IL-1 antagonism ameliorated SCC inoculation-induced mechanical allodynia in the tongue. It is well known that stimulation of P2X7R triggers activation of the NLRP3 inflammasome, resulting in the production of IL-1β [12]. Therefore, it is possible that ATP released via PANX1 in microglia may act on microglia in a paracrine manner, leading to IL-1β synthesis. IL-1β, which is synthesized in microglia in the spinal cord during joint pain, is suppressed by microglia-specific *Panx1*-deficient mice [8]. Therefore, PANX1-dependent ATP secretion causes IL-1β production in microglia following SCC inoculation. However, in vitro experiments show that P2X7R stimulation alone has little or no effect on IL-1β synthesis in microglia and priming with lipopolysaccharide, zymosan, or polyinosinic-polycytidylic acid is required for sufficient IL-1β synthesis in microglia [19]. It is assumed that microglial priming factors are synthesized in the Vc after SCC inoculation, but these factors remain unknown. Activation of microglia during SCC inoculation was observed in a limited area of the Vc and was distinct from that in the entire Vc, as seen in neuropathic pain [6,15,20]. Therefore, substances secreted by the lingual nerve from the tongue that project to a limited area in the Vc may cause microglial priming.

IL-1β has been implicated in various pain models and IL-1β can enhance neuronal activity [21]. IL-1β contributes to the phosphorylation of GluN2A and GluN2B, *N*-methyl-D-aspartate receptor (NMDAR) subunits, through the activation of Src kinase [22]. IL-1β potentiates NMDAR-mediated spontaneous excitatory synaptic currents in spinal neurons [23]. A robust Ca^2+^ influx through NMDARs leads to ERK phosphorylation [24]. Thus, inhibition of IL-1β signaling might inhibit NMDAR phosphorylation in Vc after SCC inoculation. In addition to NMDARs, α-amino-3-hydroxy-5-methyl-4-isoxazolepropionic acid receptors are also activated by IL-1β [23,25]. Accordingly, IL-1β synergistically potentiates excitatory neurotransmission.

The involvement of microglia in neuropathic pain has been clarified in several studies. In particular, the microglia-dependent pathway is specific to male animals, whereas a microglia-independent pathway has been demonstrated in female animals [26]. In addition, in clinical practice, men are known to have a higher incidence of oral cancer than women [27]. According to the abovementioned evidence, the secretion of ATP via PANX1 in microglia is likely to occur only in male rats. We could not rule out the possibility that the microglial PANX1 is involved in tongue cancer pain. We need to clarify whether there are sex-related differences in tongue cancer pain.

The limitations of our study are as follows: The study using BBG suggests the involvement of P2X7R in the Vc during SCC inoculation-induced mechanical allodynia in the tongue. However, because P2X7R is broadly expressed in the CNS, we cannot evaluate the function of P2X7R in a cell type-specific manner only by pharmacological experiments. We believe that the cells that contribute to SCC-induced mechanical allodynia in the tongue can be identified by analysis using *P2rx7*-conditional knockout mice in future studies.

Opioids have been used against cancer pain in the orofacial region. Nevertheless, the doses of opioids used in the treatment of oral cancer are higher than those used for other areas of the body and, thus, tolerance can occur easily [28]. Therefore, there is a need for new analgesics to replace opioids. It is highly possible that PANX1 is a therapeutic target for the development of appropriate drugs to prevent tongue cancer pain.

In this context, a PANX1 inhibitor could potentially be useful as a new treatment for tongue cancer pain. However, the route of administration is through the cisterna magna, and it is necessary to make a hole in the skull. It would be useful to develop a type of drug that can cross the blood-brain barrier when applied to humans.

These observations suggest that neuron-glial cell communication via PANX1 and P2X7Rs in the Vc plays a crucial role in persistent tongue pain associated with tongue SCC. 

## 4. Materials and Methods

### 4.1. Animals

Animal experiments were approved by the experimentation committee of Nihon University (protocol number: AP18DEN004-2). Animal use and care were followed by the International Association for the Study of Pain guidelines. A total of 98 male Fisher rats (weighing 200–250 g) were purchased from Japan SLC (Hamamatsu, Japan). Rats were kept at 23 ± 1 °C in 12 h light/dark cycles under specific pathogen-free conditions with free access to food and water and two rats were maintained in each cage. The number of animals was reduced for appropriate statistical analysis in this study.

### 4.2. SCC Inoculation

Cultivation of SCC cells (JCRB0231; JCRB Cell Bank, Ibaraki, Japan) and SCC inoculation in the tongue were conducted as previously described [6,29]. Briefly, SCC cells were cultured in Dulbecco’s modified Eagle’s medium (Thermo Fisher Scientific, Waltham, MA, USA) supplemented with 10% fetal bovine serum (Thermo Fisher Scientific), 100 μg/mL streptomycin (Thermo Fisher Scientific) and 100 units/mL penicillin (Thermo Fisher Scientific) at 37 °C in 5% CO_2_. SCC cells were collected from subconfluent cell culture. SCC (5  ×  10^6^ cells were suspended in 30 µL of 0.01 M PBS) cells were inoculated into the left edge of the tongue with a 26 gauge-needle under anesthesia with a mixture of butorphanol tartrate (2.5 mg/kg; Meiji Seika Pharma, Tokyo, Japan), midazolam (2.0 mg/kg; Sandoz, Tokyo, Japan) and medetomidine (0.375 mg/kg; Zenoaq, Fukushima, Japan). For the sham group, 30 µL of 0.01 M PBS was injected into the tongue.

### 4.3. Intracisternal Administration of Drugs

Intracisternal administration was performed according to a previously described method [15]. Briefly, a sterilized cannula (SP-10; Natsume, Tokyo, Japan) was placed in the cisternal magna under anesthesia with a mixture of butorphanol tartrate, midazolam and medetomidine. An osmotic pump (model 1002; Alzet, Cupertino, CA, USA) that was filled with 0.01 M PBS or 10Panx was connected to the implanted cannula. PBS, 10Panx (20 nmol/0.5 mL/h), BBG (7 pmol/0.5 mL/h), or BzATP (20 pmol/0.5 mL/h) were continuously delivered in the Vc region after SCC inoculation or PBS injection in the tongue.

### 4.4. Measurement of Head-Withdrawal Threshold 

To assess MHWT, the rats were maintained at a weak anesthesia level according to previously described criteria [29]. The left edge of the tongue (6 mm^2^, 5 mm posterior to the tip of the tongue) was pinched using digital forceps (Bioseb, Paris, France). Stimulus intensity gradually increased at a rate of 10 g/s. The MHWT was determined when the rats showed a head-withdrawal response after tongue stimulation with the lowest stimulus intensity. The cut-off value was set at 200 g to avoid tissue damage. After the MHWT measurement, the rats were anesthetized again and MHWT measurements were taken. The stimulus intensity was measured three times and the average value of the MHWT was calculated.

### 4.5. Repetitive Stimulation of the Tongue

On day 14 after PBS injection or SCC inoculation with intracisternal administration of PBS or 10Panx, the rats were anesthetized with a mixture of butorphanol tartrate, midazolam and medetomidine. The tongue was stimulated with a von Frey filament (60 g) at 1 Hz for 10 min. Five minutes after the cessation of tongue stimulation, the rats were immediately fixed with 4% paraformaldehyde (PFA). The PFA perfusion method is described in the following section. The rats with repetitive stimulation of the tongue were used to analyze the number of pERK-IR Vc neurons.

### 4.6. Immunohistochemistry

The rats were deeply anesthetized with 5% isoflurane inhalation 14 days after SCC inoculation or PBS injection and perfused transcardially with saline, followed by 0.1 M phosphate buffer (pH 7.4) containing 4% PFA. The Vc was excised and further incubated in 4% PFA for 24 h. To prevent cryolesion, Vc was further incubated with 20% sucrose in 0.01 M PBS for 24 h. The Vc slices (30 μm thick) from the brainstem section (±5 mm from the obex) were made using a microtome (SM2010R; Leica, Wetzlar, Germany). Five slices from each rat were blocked with 0.01 M PBS containing 3% normal goat serum and 0.3% Triton-X-100 for 1 h at room temperature. The slices were incubated with rabbit polyclonal anti-PANX1 antibody (1:50; Cat. MBS2001670, MYBioSource, San Diego, CA, USA), goat polyclonal anti-Iba1 antibody (1:1000; Cat. ab5076, Abcam, Cambridge, MA, USA), mouse monoclonal anti-GFAP antibody (1:1000; Cat. MAB360, Merck Millipore, Billerica, MA, USA), or mouse monoclonal anti-NeuN antibody (1:100; Cat. MAB377; Merck Millipore) for 2 days at 4 °C. After washing with 0.01 M PBS 10 min three times, the slices were further incubated with secondary antibody conjugated with Alexa Fluor 488 or Alexa Fluor 555 (1:1000; Thermo Fisher Scientific) for 2 h at room temperature. After gentle wash with 0.01 M PBS, the slices were mounted on an anti-fading medium (PermaFluor Aqueous Mounting Medium, Thermo Fisher Scientific). The immunofluorescent signal, which was more than twice as strong as its background, was captured by a BZ-9000 (Keyence, Osaka, Japan). The number of PANX1-IR cells in the section (727 μm × 547 μm) was counted. 

To detect pERK-IR neurons, Vc slices were incubated with mouse monoclonal anti-pERK antibody (1:1000; Cat. No. 9106, Cell Signaling, Danvers, MA, USA) for 2 days at 4 °C. The slices were incubated with biotinylated anti-mouse secondary antibody (1:600; Cat. BA-1000-1.5; Vector Laboratories, Burlingame, CA, USA) for 2 h at room temperature, followed by incubation with avidin-biotin complex solution (Cat. No. PK-4000, Vector Laboratories) for 2 h at room temperature. pERK-IR neurons were visualized by reaction with 0.05 M Tris buffer (pH 7.6) containing 0.02% 3,3′-diaminobenzidine tetrahydrochloride, 0.5% nickel ammonium sulfate and 0.03% H_2_O_2_ for 5–15 s. The Vc slices were mounted on EUKITT (O. KINDLAR, Freiburg, Germany). Images were captured using a camera (DP70; Olympus, Tokyo, Japan) with a microscope (BX51, Olympus). The number of pERK-IR neurons in the section (898 μm × 676 μm) was counted. 

### 4.7. Single Neuron Recording

Rats (PBS + PBS, *n* = 5; SCC + PBS, *n* = 6; SCC + 10Panx, *n* = 4) were anesthetized with a mixture of butorphanol tartrate, midazolam and medetomidine and placed on a stereotaxic instrument. The trachea and femoral vein were cannulated to supply isoflurane (2%) and rocuronium bromide (0.6 mg/kg/h, i.v., MSD, Tokyo, Japan) during surgery and recording. Laminectomy was conducted in the brainstem region, including the Vc and the upper cervical spinal cord. Photomicrographs of the brainstem surface were obtained to identify the recording position. Tungsten microelectrodes (FHC Instruments, Bowdoin, ME, USA) were inserted into the dorsal portion of the Vc (~1500 μm depth from the surface) and neuronal activity was recorded using Spike II software (CED1401; CED, Cambridge, UK). The RFs were mapped from the neuronal responses obtained from gentle pressure stimulation of the tongue surface. Then, brush stimuli, non-noxious and noxious pressure and pinch stimuli were applied to the center of the RFs. The percentage of RFs in the entire tongue area was calculated. After the cessation of recordings, the recording points were marked by applying direct current (20 mA, 10 s) and potassium chloride (2 M) was injected through the femoral vein to euthanize the rats. The brainstems were removed and the tissues were soaked in 4% PFA for 1 week and the sagittal sections of the brainstem were cut and stained with hematoxylin-eosin to check the recording site. The recording sites were plotted on the transverse plane of the brainstem images. Spike frequency was analyzed using Spike II software (CED) and the mean spike frequencies were calculated.

### 4.8. Statistical Analysis

Drug selection was performed blindly by the researcher. Data are shown as mean ± standard error of the mean (SEM). The normality of the sample data was assessed using the Shapiro–Wilk test. Statistical analyses were performed using a two-way repeated-measures analysis of variance (ANOVA) followed by the Bonferroni’s test, one-way ANOVA followed by Tukey’s or Dunnett’s test, or unpaired Student’s *t*-test using the GraphPad Prism 7 software program (GraphPad Software Inc., San Diego, CA, USA), as appropriate. Differences were considered significant at *p* < 0.05. The number (*n*) and statistical methods are indicated in the figure legends.

## Data Availability

MDPI Research Data Policies.

## Abbreviations

SCC	squamous cell carcinoma
QOL	quality of life
TG	trigeminal ganglion
Vc	trigeminal spinal subnucleus caudalis
CNS	central nervous system
PANX1	pannexin-1
ATP	adenosine triphosphate
IL	interleukin
MHWT	mechanical head-withdrawal threshold
PBS	phosphate-buffered saline
IR	immunoreactive
Iba1	ionized calcium-binding adapter molecule 1
GFAP	glial fibrillary acidic protein
NeuN	neuronal nuclei
RFs	receptive fields
WDR	wide dynamic range
pERK	phosphorylated extracellular signal-regulated kinase
BBG	Brilliant Blue G
NLRP3	nod-like receptor family pyrin domain containing 3
NMDAR	*N*-methyl-_D_-aspartate receptor
PFA	paraformaldehyde
SEM	standard error of the mean
ANOVA	analysis of variance

## References

[1] Vigneswaran N., Williams M.D. (2014). Epidemiologic trends in head and neck cancer and aids in diagnosis. Oral Maxillofac. Surg. Clin. N. Am..

[2] Viet C.T., Schmidt B.L. (2012). Biologic mechanisms of oral cancer pain and implications for clinical therapy. J. Dent. Res..

[3] Ye Y., Dang D., Zhang J., Viet C.T., Lam D.K., Dolan J.C., Gibbs J.L., Schmidt B.L. (2011). Nerve growth factor links oral cancer progression, pain, and cachexia. Mol. Cancer Ther..

[4] Ishimoto S., Wada K., Tanaka N., Yamanishi T., Ishihama K., Aikawa T., Okura M., Nakajima A., Kogo M., Kamisaki Y. (2012). Role of endothelin receptor signalling in squamous cell carcinoma. Int. J. Oncol..

[5] Chichorro J.G., Zampronio A.R., Rae G.A. (2006). Endothelin ET(B) receptor antagonist reduces mechanical allodynia in rats with trigeminal neuropathic pain. Exp. Biol. Med. (Maywood).

[6] Tamagawa T., Shinoda M., Honda K., Furukawa A., Kaji K., Nagashima H., Akasaka R., Chen J., Sessle B.J., Yonehara Y. (2016). Involvement of Microglial P2Y12 Signaling in Tongue Cancer Pain. J. Dent. Res..

[7] Yeung A.K., Patil C.S., Jackson M.F. (2020). Pannexin-1 in the CNS: Emerging concepts in health and disease. J. Neurochem..

[8] Mousseau M., Burma N.E., Lee K.Y., Leduc-Pessah H., Kwok C.H.T., Reid A.R., O’Brien M., Sagalajev B., Stratton J.A., Patrick N. (2018). Microglial pannexin-1 channel activation is a spinal determinant of joint pain. Sci. Adv..

[9] Burma N.E., Bonin R.P., Leduc-Pessah H., Baimel C., Cairncross Z.F., Mousseau M., Shankara J.V., Stemkowski P.L., Baimoukhametova D., Bains J.S. (2017). Blocking microglial pannexin-1 channels alleviates morphine withdrawal in rodents. Nat. Med..

[10] Pelegrin P., Surprenant A. (2006). Pannexin-1 mediates large pore formation and interleukin-1beta release by the ATP-gated P2X7 receptor. EMBO J..

[11] Sorge R.E., Trang T., Dorfman R., Smith S.B., Beggs S., Ritchie J., Austin J.S., Zaykin D.V., Vander Meulen H., Costigan M. (2012). Genetically determined P2X7 receptor pore formation regulates variability in chronic pain sensitivity. Nat. Med..

[12] Di Virgilio F., Dal Ben D., Sarti A.C., Giuliani A.L., Falzoni S. (2017). The P2X7 Receptor in Infection and Inflammation. Immunity.

[13] Noma N., Tsuboi Y., Kondo M., Matsumoto M., Sessle B.J., Kitagawa J., Saito K., Iwata K. (2008). Organization of pERK-immunoreactive cells in trigeminal spinal nucleus caudalis and upper cervical cord following capsaicin injection into oral and craniofacial regions in rats. J. Comp. Neurol..

[14] Nakaya Y., Tsuboi Y., Okada-Ogawa A., Shinoda M., Kubo A., Chen J.Y., Noma N., Batbold D., Imamura Y., Sessle B.J. (2016). ERK-GluR1 phosphorylation in trigeminal spinal subnucleus caudalis neurons is involved in pain associated with dry tongue. Mol. Pain.

[15] Asano S., Hayashi Y., Iwata K., Okada-Ogawa A., Hitomi S., Shibuta I., Imamura Y., Shinoda M. (2020). Microglia-Astrocyte Communication via C1q Contributes to Orofacial Neuropathic Pain Associated with Infraorbital Nerve Injury. Int. J. Mol. Sci..

[16] Kiyomoto M., Shinoda M., Okada-Ogawa A., Noma N., Shibuta K., Tsuboi Y., Sessle B.J., Imamura Y., Iwata K. (2013). Fractalkine signaling in microglia contributes to ectopic orofacial pain following trapezius muscle inflammation. J. Neurosci..

[17] Tsuda M., Inoue K., Salter M.W. (2005). Neuropathic pain and spinal microglia: A big problem from molecules in “small” glia. Trends Neurosci..

[18] Huang Y.J., Maruyama Y., Dvoryanchikov G., Pereira E., Chaudhari N., Roper S.D. (2007). The role of pannexin 1 hemichannels in ATP release and cell-cell communication in mouse taste buds. Proc. Natl. Acad. Sci. USA.

[19] Ferrari D., Villalba M., Chiozzi P., Falzoni S., Ricciardi-Castagnoli P., Di Virgilio F. (1996). Mouse microglial cells express a plasma membrane pore gated by extracellular ATP. J. Immunol..

[20] Shibuta K., Suzuki I., Shinoda M., Tsuboi Y., Honda K., Shimizu N., Sessle B.J., Iwata K. (2012). Organization of hyperactive microglial cells in trigeminal spinal subnucleus caudalis and upper cervical spinal cord associated with orofacial neuropathic pain. Brain Res..

[21] Goncalves Dos Santos G., Delay L., Yaksh T.L., Corr M. (2019). Neuraxial Cytokines in Pain States. Front. Immunol..

[22] Viviani B., Bartesaghi S., Gardoni F., Vezzani A., Behrens M.M., Bartfai T., Binaglia M., Corsini E., Di Luca M., Galli C.L. (2003). Interleukin-1beta enhances NMDA receptor-mediated intracellular calcium increase through activation of the Src family of kinases. J. Neurosci..

[23] Kawasaki Y., Zhang L., Cheng J.K., Ji R.R. (2008). Cytokine mechanisms of central sensitization: Distinct and overlapping role of interleukin-1beta, interleukin-6, and tumor necrosis factor-alpha in regulating synaptic and neuronal activity in the superficial spinal cord. J. Neurosci..

[24] Zhou X., Moon C., Zheng F., Luo Y., Soellner D., Nunez J.L., Wang H. (2009). N-methyl-D-aspartate-stimulated ERK1/2 signaling and the transcriptional up-regulation of plasticity-related genes are developmentally regulated following in vitro neuronal maturation. J. Neurosci. Res..

[25] Lai A.Y., Swayze R.D., El-Husseini A., Song C. (2006). Interleukin-1 beta modulates AMPA receptor expression and phosphorylation in hippocampal neurons. J. Neuroimmunol..

[26] Sorge R.E., Mapplebeck J.C., Rosen S., Beggs S., Taves S., Alexander J.K., Martin L.J., Austin J.S., Sotocinal S.G., Chen D. (2015). Different immune cells mediate mechanical pain hypersensitivity in male and female mice. Nat. Neurosci..

[27] El-Naggar A.K., Chan J.C.K., Grandis J.R., Takata T., Slootweg P.J. (2017). WHO Classification of Head and Neck Tumours.

[28] Mercadante S., Dardanoni G., Salvaggio L., Armata M.G., Agnello A. (1997). Monitoring of opioid therapy in advanced cancer pain patients. J. Pain Symptom Manag..

[29] Furukawa A., Shinoda M., Kubo A., Honda K., Akasaka R., Yonehara Y., Iwata K. (2018). Endothelin Signaling Contributes to Modulation of Nociception in Early-stage Tongue Cancer in Rats. Anesthesiology.

